# The Impact of Local Ablative Therapies as Bridging Treatment on Overall Survival Following Liver Transplantation in Patients with HCC

**DOI:** 10.3390/cancers17203393

**Published:** 2025-10-21

**Authors:** Laura Schwenk, Felix Dondorf, Oliver Rohland, Aladdin Ali-Deeb, Utz Settmacher, Falk Rauchfuß

**Affiliations:** 1 Department of General, Visceral and Vascular Surgery, Jena University Hospital, 07747 Jena, Germany; felix.dondorf@med.uni-jena.de (F.D.); oliver.rohland@med.uni-jena.de (O.R.); aladdin.ali-deeb@med.uni-jena.de (A.A.-D.); utz.settmacher@med.uni-jena.de (U.S.); falk.rauchfuss@med.uni-jena.de (F.R.); 2 Comprehensive Cancer Center Central Germany-Campus Jena, 07747 Jena, Germany; 3 Interdisciplinary Center for Clinical Research (IZKF), Jena University Hospital, 07747 Jena, Germany

**Keywords:** hepatocellular carcinoma, neoadjuvant therapy, SIRT, TACE, liver resection, liver transplantation, OS, transplantation criteria, tumor burden

## Abstract

**Simple Summary:**

The use of neoadjuvant therapies in patients with hepatocellular carcinoma prior to liver transplantation has gained increasing relevance in recent years, yet evidence regarding their impact on post-transplant outcomes remains limited. This study aimed to assess the effect of neoadjuvant therapies on overall survival following liver transplantation in patients with hepatocellular carcinoma and to compare outcomes across treatment modalities with respect to tumor burden. A total of 107 patients were analyzed, including 90 who underwent neoadjuvant treatment. Therapeutic strategies comprised SIRT, TACE, liver resection, and combined SIRT/TACE. Neoadjuvant therapy was associated with a significant survival benefit after liver transplantation. TACE demonstrated the greatest efficacy in patients meeting established transplant criteria, typically characterized by lower tumor burden, whereas SIRT conferred superior benefit in patients with higher tumor burden or those beyond conventional listing criteria.

**Abstract:**

Background: The use of neoadjuvant therapies in patients with hepatocellular carcinoma prior to liver transplantation has gained increasing popularity in recent years. To date, there are only limited data investigating the impact of neoadjuvant therapy on post-transplant survival. Methods: In this retrospective study, we evaluated patients with hepatocellular carcinoma who underwent deceased donor or living donor liver transplantation at Jena University Hospital between 2019 and 2023. Comprehensive clinical and pathological variables were systematically analyzed, including correlations between neoadjuvant therapy use, tumor burden and overall survival. Survival outcomes were estimated using the Kaplan–Meier method. Results: A total of 107 patients were included in the analysis, of whom 90 received neoadjuvant therapy prior to transplantation. Treatment modalities comprised SIRT, TACE, liver resection and combined SIRT and TACE. The 1-, 3-, and 5-year OS rates following transplantation were 93.5%, 82.2%, and 79.4%, respectively. Recurrence-free survival at 1, 3, and 5 years was 91.6%, 85.0%, and 83.2%, respectively. Among the various neoadjuvant strategies, SIRT and TACE yielded the highest OS rates. Patients listed outside the transplantation criteria (Milan, UCSF, up-to-seven) at the time of initial diagnosis who underwent SIRT had significantly better OS than those outside the criteria who underwent TACE. In contrast, among patients within the Milan, UCSF and up-to-seven criteria, TACE was associated with superior OS compared with SIRT. Conclusion: The use of neoadjuvant therapies confers a significant survival benefit following liver transplantation in patients with HCC. TACE appears to be most suitable for patients listed within established transplantation criteria, who consequently have a lower tumor burden. In contrast, SIRT is more beneficial for patients with a higher tumor burden and those beyond standard transplantation criteria. A limitation of our study, however, is that the included SIRT cohort comprised only 24 patients, and TACE was preferentially performed in patients with a lower tumor burden, which means that a selection bias cannot be fully excluded. Overall, further studies are required to define the optimal bridging strategies.

## 1. Introduction

Hepatocellular carcinoma (HCC) is one of the leading causes of cancer-related mortality worldwide and represents a major contributor to death in patients with liver cirrhosis [1].

Over the past decades, liver transplantation (LT) has emerged as one of the most successful curative treatment options for patients with HCC. This is particularly relevant given that the majority of patients present with underlying cirrhosis at the time of diagnosis, rendering them ineligible for surgical resection. Initially, suboptimal patient selection, often due to advanced tumor burden and inaccurate tumor characterization, resulted in poor long-term survival outcomes and high post-transplant recurrence rates [2].

The introduction of transplantation criteria has led to two key outcomes. First, it enables more effective management of the persistent organ shortage, as only a minority of patients meet the criteria at initial diagnosis, leaving most with limited access to timely liver transplantation. Second, the application of these criteria has significantly improved post-transplant oncological outcomes; following the implementation of the Milan criteria (MC) in 1996, which demonstrated a 4-year overall survival rate of 75% and a recurrence-free survival rate of 83% [3,4].

Since then, several other selection systems have been established, including the UCSF (University of California San Francisco) criteria [5] and the up-to-seven criteria [6], all aiming to refine patient eligibility for transplantation.

Established neoadjuvant therapies (NTs) include, among others, transarterial chemoembolization (TACE) [7], selective internal radiation therapy (SIRT) [8], radiofrequency ablation (RFA) [9], microwave ablation (MWA) [10], surgical resection, and radiation therapy [11]. To date, only a limited number of studies have evaluated the impact of these NT on post-transplant survival outcomes. Notably, comparative data assessing the relative efficacy of these therapeutic approaches remain limited, and consensus on a standardized treatment algorithm has yet to be established.

The aim of this study was to evaluate the impact of NT on overall survival following liver transplantation in patients with HCC and to compare the outcomes of different therapeutic approaches with respect to tumor burden.

## 2. Materials and Methods

Data were evaluated from patients who underwent deceased donor or living donor liver transplantation for HCC at the Jena University Hospital between 2019 and 2023.

The following parameters were evaluated: overall survival (OS), disease-free survival (DFS), and recurrence rate. Patient diagnoses, eligibility criteria for liver transplantation (Milan cirteria, UCSF-criteria, up to seven-criteria), NT, and type of transplantation, tumor-specific characteristics such as tumor size and tumor classification, as well as general patient data in the context of clinical, surgical, and pathological findings, were extracted from the hospital’s SAP database (SAP Global Corporate Affairs, Walldorf, Germany). Selected patient data are presented as means and ranges. The tumor burden was defined based on the number of HCC lesions.

Survival studies were determined using the Kaplan–Meier method and group differences in overall survival were assessed using the log-rank test. Overall survival was defined as the duration between initial diagnosis and death. To identify independent prognostic factors for overall survival, a multivariate Cox proportional hazards regression analysis was performed. Variables included in the model were selected based on clinical relevance and univariate analysis results. Hazard ratios (HRs) with 95% confidence intervals (CIs) and corresponding *p*-values were calculated. The proportional hazards assumption was verified using log-minus-log plots and time-dependent covariates, where applicable. For all analyses, SPSS statistics (Version: 29.0.1.0., IBM Corp., Armonk, NY, USA) and Microsoft Excel (Version: 16.16.27., Microsoft Corporation, Redmond, WA, USA) were used.

To access the current main studies regarding liver transplantation for HCC and NT, we performed literature research and discussed this in the context of our results using the following search keywords: “liver transplantation”, “hepatocellular carcinoma”, “neoadjuvant therapy” and “overall survival”. The electronic databases included the following: PubMed, Google Scholar and MEDLINE.

## 3. Results

A total of 107 patients were included in the analysis. In four cases, the postoperative histopathological examination revealed a diagnosis of combined hepatocellular-cholangiocarcinoma. These four patients were included in the analysis. 68 patients (63.6%) underwent deceased donor liver transplantation, while 39 patients (36.4%) received a living donor liver transplant.

The mean age at diagnosis was 60 years (range: 24–72), and the mean age at transplantation was 61 years (range: 24–73). 97 patients (90.7%) were male, and 10 patients (9.3%) were female. Patient characteristics are summarized in Table 1. The mean diameter of the largest HCC lesion at diagnosis was 3.7 cm (range: 0.5–14 cm). Preoperative imaging detected a single HCC lesion in 42 patients (39.3%), two lesions in 30 patients (28%), three lesions in 12 patients (11.2%), and four lesions in 5 patients (4.7%). In 18 patients (16.8%), five or more lesions were identified (Table 1).

**Figure 1:**
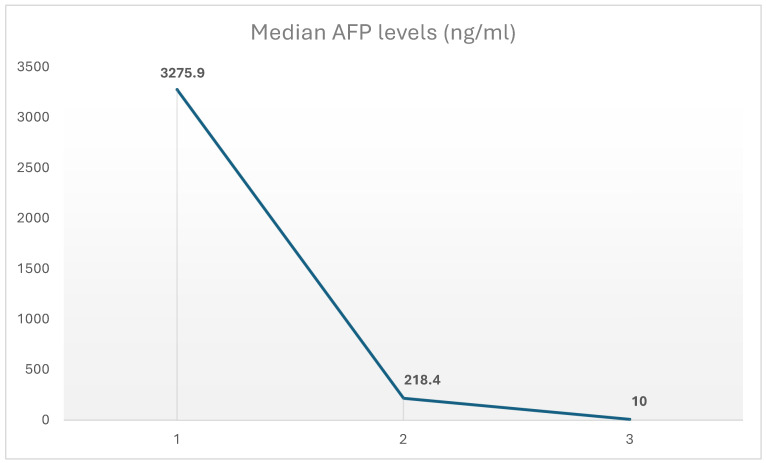
Median AFP levels. Abbreviations: AFP: Alpha-fetoprotein.

The median alpha-fetoprotein (AFP) levels at initial diagnosis, after NT and postoperatively were 3275.9 ng/mL, 218.4 ng/mL and 10 ng/mL, respectively (see Figure 1).

### 3.1. Neoadjuvant Therapy

A total of 90 patients received NT. The neoadjuvant treatment strategies are summarized in Table 1. The applied modalities included SIRT in 24 patients (22.4%), TACE in 49 patients (45.8%), liver resection in 3 patients (2.8%) and a combination of SIRT and TACE in 7 patients (6.5%). Two patients underwent radiofrequency ablation, three patients received external beam radiation therapy and two patients received chemotherapy. Due to the limited sample size, they were grouped together under the category “Other”.

A radiographic response to NT was observed in 57 cases (53.3%). Seven patients showed no response or progressive disease during neoadjuvant treatment (6.5%). In 26 patients (24.3%), no follow-up imaging was performed after NT, as they underwent transplantation prior to the planned follow-up assessment.

At the time of initial diagnosis, 62 patients (57.9%) were listed within the Milan criteria, whereas 45 patients (42.1%) were listed outside.

71 patients (66.4%) met the UCSF criteria and 76 patients (71%) met the up-to-seven criteria (Table 2).

A comparison between tumor burden and the applied NT is presented in Table 3.

Post-transplant histopathological analysis revealed complete regression of HCC lesions in 8 patients. Residual tumor burden was observed as follows: 47 patients had one lesion, 17 patients had two lesions, 14 patients had three lesions, 3 patients had four lesions, and 18 patients had five or more lesions. Compared to the preoperative tumor count, 83 patients (77.6%) demonstrated either regression or stable tumor burden postoperatively.

The mean diameter of the largest HCC lesion in the pathological specimen after transplantation was 3.0 cm (range: 0–14.5 cm). Among patients who received neoadjuvant therapy, 68 patients (63.6%) exhibited tumor regression or stable tumor burden postoperatively, and 59 patients (55.1%) showed regression or stability with respect to the maximum tumor diameter.

### 3.2. Survival

A total of 29 patients (27.1%) died during the follow-up period. The 1-, 3-, and 5-year overall survival (OS) rates post-transplantation were 93.5%, 82.2%, and 79.4%, respectively. The median follow-up duration was 4.8 years.

**Figure 2:**
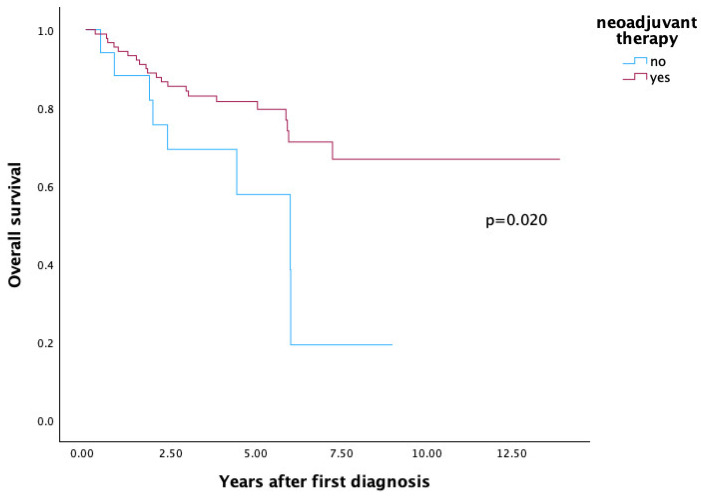
Impact of Neoadjuvant Therapy on Overall Survival.

Patients who received neoadjuvant therapy demonstrated significantly improved overall survival compared to those who did not (66.8% vs. 19.3%, *p* = 0.020) (see Figure 2).

### 3.3. Comparison of Neoadjuvant Therapies Regarding Overall Survival

Among the different NT, SIRT and TACE showed the best overall survival outcomes (*p* = 0.021).

**Figure 3:**
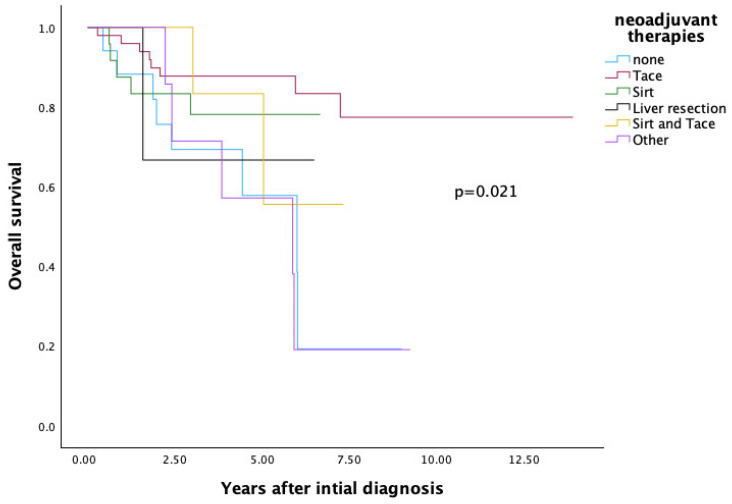
Comparison of Neoadjuvant Therapies Regarding Overall Survival.

The OS was 19.3% for patients without NT, 78.1% for those receiving SIRT alone, 77.4% for TACE alone, 66.7% for liver resection, 55.6% for the combined SIRT and TACE group and 19% and 19% for other therapies (see Figure 3).

### 3.4. SIRT and TACE—Comparison of Overall Survival Based on Transplantation Criteria

#### 3.4.1. Milan Criteria

The overall survival rate for patients who did not receive SIRT or TACE (n = 34) was 29.5%.

Among patients listed within the Milan criteria at the time of initial diagnosis and treated with either TACE or SIRT, overall survival was 82.7% and 83.3%, respectively.

In comparison, for patients listed outside the Milan criteria and treated with TACE or SIRT, survival rates were 12.6% and 76.4%, respectively.

**Figure 4:**
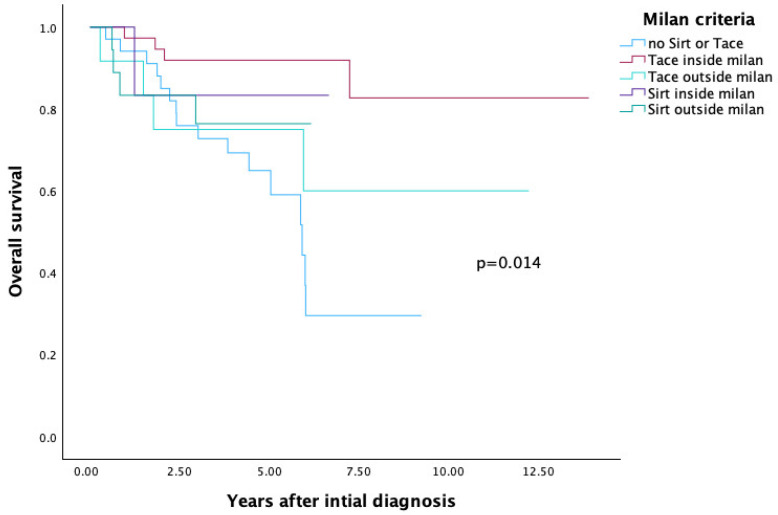
Milan Criteria—Overall Survival.

These differences were statistically significant (*p* = 0.014) (see Table 4 and Figure 4).

#### 3.4.2. UCSF Criteria

A significant correlation was also observed between overall survival and the UCSF criteria (*p* = 0.002).

Once again, patients who did not receive SIRT or TACE prior to transplantation had an overall survival rate of 29.5%.

Among those within the UCSF criteria at initial diagnosis and bridged with TACE or SIRT, overall survival reached 85.6% and 75%, respectively.

**Figure 5:**
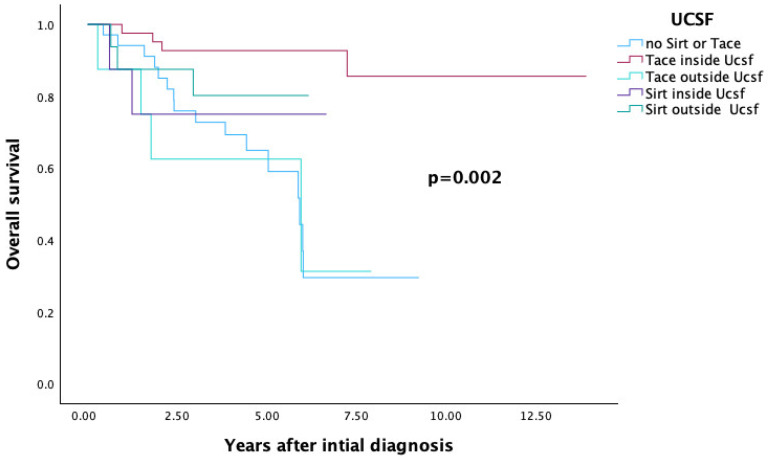
UCSF Criteria—Overall Survival.

In contrast, patients outside the UCSF criteria who received TACE or SIRT had survival rates of 31.3% and 80.2%, respectively (see Table 4 and Figure 5).

#### 3.4.3. Up-to-Seven Criteria

A significant correlation was also found between overall survival and the up-to-seven criteria (*p* = 0.001).

Patients who did not undergo SIRT or TACE before transplantation had an overall survival rate of 29.5%. Among patients listed within the up-to-seven criteria at diagnosis and bridged with TACE or SIRT, overall survival was 85.3% and 80%, respectively.

**Figure 6:**
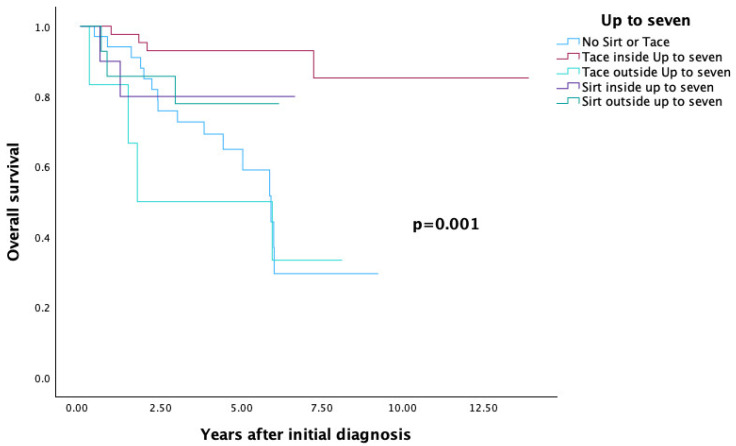
Up to seven Criteria—Overall Survival.

For those outside these criteria, survival was 33.3% for TACE and 77.9% for SIRT (see Table 4 and Figure 6).

### 3.5. Multivariate Analysis

The results of the multivariate Cox regression analysis are presented in Table 5. In the multivariate analysis, negative AFP levels following neoadjuvant therapy, negative postoperative AFP levels, administration of neoadjuvant therapy, and radiographic response to neoadjuvant therapy were identified as significant independent prognostic factors for overall survival.

### 3.6. Recurrence Rate

By the final follow-up date, 19 patients (82.2%) had confirmed HCC recurrence. The median time from transplantation to recurrence was 19.3 months. Recurrence-free survival rates at 1, 3, and 5 years were 91.6%, 85%, and 83.2%, respectively. The most common site of recurrence was the transplanted liver (see Table 1).

## 4. Discussion

In our study, we demonstrated that the use of NT confers a significant survival benefit in patients with HCC following liver transplantation. Furthermore, we were able to demonstrate for the first time that TACE may be more appropriately applied in patients with a lower tumor burden and in those who meet established transplantation criteria. In contrast, SIRT may provide a survival advantage for patients with a higher tumor burden and for those beyond standard transplantation criteria.

It is undeniable that since the introduction of the Milan Criteria, excellent 5-year overall survival rates of 60–70% following liver transplantation have been observed in patients with HCC [12,13]. However, a major drawback is the significant increase in waiting times on transplant lists over the years. This is largely attributable to the persistent shortage of donor organs, which stands in stark contrast to the steadily rising demand for liver transplants. For HCC patients who fall outside established transplant criteria, this situation translates into a progressively diminishing likelihood of receiving a donor organ. Prolonged waiting times frequently result in tumor progression while on the list, with reported annual dropout rates due to tumor progression reaching approximately 30–40% [14].

Several authors have demonstrated that bridging or downstaging therapies can effectively delay tumor progression, thereby significantly reducing the risk of dropout from the liver transplant waiting list [15,16].

Lai et al. showed in their intention-to-treat analysis that the risk of waitlist dropout due to tumor progression could be reduced by 34–49% through the application of neoadjuvant treatment strategies [17].

Similarly, Xing et al. demonstrated that neoadjuvant therapy has a significant effect on progression-related waitlist dropout. In their study, they analyzed 205 patients who met the Milan criteria and subsequently underwent LT. Among these, 111 patients received neoadjuvant therapy, while 94 did not. Their results indicated that patients who received bridging therapy had a significantly lower dropout rate (2.58%) compared to those without bridging therapy (8.18%). Moreover, the authors reported that the median overall survival after LT was 86.4 months in the group that received neoadjuvant therapy, compared to 68.9 months in the group without (*p* = 0.01). In multivariate analysis, both longer waiting times (*p* = 0.005) and neoadjuvant therapy (*p* = 0.005) were identified as independent predictors of post-transplant survival [18].

The use of neoadjuvant treatment regimens has become increasingly established in many transplant centers in recent years for the management of patients with HCC [19]. Although bridging therapy is recommended in current clinical guidelines, there remains no clear consensus on the most appropriate modality. Consequently, the choice of bridging or downstaging therapy is typically made on an individualized basis by the respective transplant center, depending on patient-specific factors, including contraindications and comorbidities [20].

The finding that patients receiving neoadjuvant therapy prior to planned LT have a survival advantage compared to those who do not has been consistently reported by several authors in recent years [21,22,23,24,25].

The question of which neoadjuvant therapy provides the most favorable overall survival outcomes following liver transplantation remains a topic of ongoing discussion.

Several authors have reported that both TACE and SIRT yield comparable post-transplant survival rates, suggesting that the type of neoadjuvant therapy may not have a substantial impact on overall survival after LT [26,27].

In contrast, several publications in the literature support either TACE [24] or SIRT [28,29] as the superior therapeutic option.

A recently published meta-analysis by Alcantara et al. demonstrated that SIRT was associated with a significant survival advantage over TACE (*p* = 0.0009) and provided superior locoregional disease control [30].

In our analysis, we were also able to demonstrate that undergoing neoadjuvant therapy in patients with HCC leads to a significantly improved overall survival compared to patients who did not receive NT (66.8% vs. 19.3%, *p* = 0.020).

In a direct comparison of different NT modalities, TACE (77.4%) and SIRT (78.1%) yielded the best overall survival outcomes following LT.

A limitation of our study to be noted is that TACE was predominantly applied to patients with a lower tumor burden within our cohort, which introduces a potential bias.

Overall, 49 patients received TACE. At the time of initial diagnosis (prior to NT), 37 of these patients met the Milan criteria, 41 met the UCSF criteria, and 43 fulfilled the Up-to-seven criteria. In comparison, 24 patients underwent SIRT, of whom 6 were within Milan, 8 within UCSF, and 10 within the Up-to-seven criteria at initial diagnosis (prior to NT). This suggests that TACE was preferentially used in patients with a lower tumor burden (smaller tumors and/or fewer tumor lesions). Based on this observation, it can be assumed that patients pretreated with TACE would inherently be expected to have better post-LT survival due to their lower tumor burden.

Therefore, it is remarkable that patients receiving SIRT, who formally presented with a higher tumor burden, achieved a marginally improved overall survival compared to those treated with TACE (78.1% vs. 77.4%).

This finding was further supported by survival comparisons stratified according to transplantation criteria. Patients listed outside the transplant criteria (Milan, UCSF, Up-to-seven) at initial diagnosis who received SIRT demonstrated significantly better overall survival compared to those outside the criteria who underwent TACE.

Among patients within the criteria, TACE showed improved overall survival compared to SIRT within the UCSF criteria (85.6% vs. 75%) and the Up-to-seven criteria (85.3% vs. 80%).

Although neoadjuvant therapies are widely used and our study demonstrated a significant survival benefit associated with their application, further prospective studies with larger patient cohorts are needed to better define optimal and individualized treatment strategies. Future research should refine and personalize bridging approaches for different HCC subgroups. Multicenter prospective cohorts and randomized controlled trials are essential to validate the observed survival benefits of various neoadjuvant modalities. Additionally, integrating biomarkers and molecular profiling could enhance the understanding of tumor biology and treatment response, enabling a more precise, patient-tailored selection of bridging strategies based on tumor burden and transplant eligibility.

## 5. Conclusions

The use of neoadjuvant therapies confers a significant survival benefit following liver transplantation in patients with HCC. Overall, based on these findings, it may be concluded that TACE may be more appropriately utilized in patients with a lower tumor burden and those who meet established transplantation criteria.

In contrast, SIRT may be more beneficial in terms of survival for patients with an elevated tumor burden and those beyond standard transplantation criteria.

A limitation of our study, however, is that the included SIRT cohort comprised only 24 patients, and TACE was preferentially performed in patients with a lower tumor burden, which means that a selection bias cannot be fully excluded.

Overall, further studies are required to define the optimal bridging strategies.

## Figures and Tables

**Table 1:** Characteristics of patients.

Variables	Entire Cohort (*n* = 107)
**Age at the time of first diagnosis** (years, mean, range)	60 (24–72)
**Age at the time of liver transplantation** (years, mean, range)	61 (24–73)
**Gender** (Male, Female, n (%))	97 (90.7), 10 (9.3)
**BMI** (kg/m^2,^ mean, range)	28 (19.5–37.6)
**Transplantation** (LDLT, DDLT,n (%))	39 (36.4), 68 (63.6)
**Liver Cirrhosis** (yes, n (%))	99 (92.5)
**Child** (none, A,B,C, n(%))	8 (7.5), 52 (48.6), 31 (29), 16 (15)
**Nutritional toxic liver cirrhosis** (yes, n (%))	70 (65.4)
**Diagnosis** (n (%))	
HCC	103 (96.3)
HCC-iCCA	4 (3.7)
**T Stage** (n (%))	
T0	18 (16.8)
T1	30 (28)
T2	49 (45.8)
T3	7 (6.5)
T4	2 (1.9)
**Relapse** (n (%))	
Yes	19 (17.8)
No	88 (82.2)
**Site of recurrence**	
Adrenal gland	1
Lung	4
Lymph node	2
Liver	7
Bone	4
Brain	1
**Neoadjuvant therapy** (n (%))	
Yes	90 (84.1)
No	17 (15.9)
**Neoadjuvant therapy** (n (%))	
none	17 (15.9)
SIRT	24 (22.4)
TACE	49 (45.8)
Liver resection	3 (2.8)
SIRT and TACE	7 (6.5)
Other:	7 (6.5)
RFA	2
Radiatio	3
Chemotherapy	2
**Response to neoadjuvant therapy** (n (%))	
Yes	57 (53.3)
No	7 (6.5)
No follow-up imaging	26 (24.3)
No neoadjuvant therapy	17 (15.9)
**Number of lesions** (n (%))	
at time of first diagnosis	
1	42 (39.3)
2	30 (28)
3	12 (11.2)
4	5 (4.7)
≥5	18 (16.8)
	
postoperative (histopathological examination)	
0	8 (7.5)
1	47 (43.9)
2	17 (15.9)
3	14 (13.1)
4	3 (2.8)
≥5	18 (16.8)
**Largest tumor diameter** (cm, mean, range)	
at time of first diagnosis	3.7 (0.5–14)
postoperative histopathological examination	3 (0–14.5)
**AFP at initial diagnosis (n)**	
Elevated	66
Negative	41
	
**AFP after neoadjuvant therapy (n)**	
Elevated	50
Negative	49
Not available	8
	
**AFP postoperatively (n)**	
Elevated	17
Negative	83
Not available	7

Abbreviations: BMI: Body Mass Index; LDLT: living donor liver transplantation; DDLT: deceased donor liver transplantation; HCC: Hepatocellular Carcinoma; HCC-iCCA: combined hepatocellular carcinoma/cholangiocarcinoma; T Stage: Tumor stage; SIRT: selective internal radiation therapy; TACE: Transarterial chemoembolization; RFA: Radiofrequency Ablation; AFP: Alpha-fetoprotein.

**Table 2:** Transplantation criteria.

Transplantation Criteria	Entire Cohort (*n* = 107)
**Milan at time of first diagnosis n (%)**	
Inside	62 (57.9)
Outside	45 (42.1)
**Milan after neoadjuvant therapy n (%)**	
Inside	66 (61.7)
Outside	41 (38.3)
**UCSF at time of first diagnosis n (%)**	
Inside	71 (66.4)
Outside	36 (33.6)
**USCSF after neoadjuvant therapy n (%)**	
Inside	75 (70.1)
Outside	32 (29.9)
**Up to seven at time of first diagnosis n (%)**	
Inside	76 (71)
Outside	31 (29)
**Up to seven after neoadjuvant therapy n (%)**	
Inside	82 (76.6)
Outside	25 (23.4)

Abbreviations: UCSF: University of California San Francisco.

**Table 3:** Comparison between tumor burden and the applied neoadjuvant therapy.

**Neoadjuvant** **t** **herapy**	**Number of patients outside the Milan criteria** **(at the time of pre-transplant evaluation)/(based on postoperative histopathological findings)**	**Number of patients inside the Milan criteria** **(at the time of pre-transplant evaluation)/(based on postop-erative histopathological find-ings)**
None	9/11	8/6
TACE	12/15	37/34
SIRT	18/12	6/12
Liver resection	1/0	2/3
SIRT and TACE	3/1	4/6
Other	2/2	5/5
**Neoadjuvant therapy**	**Number of patients outside the UCSF criteria** **(at the time of pre-transplant evaluation)/(based on post-operative histopathological findings)**	**Number of patients inside the UCSF criteria** **(at the time of pre-transplant evaluation)/(based on postoperative histopathological findings)**
None	7/9	10/8
TACE	8/11	41/38
SIRT	16/9	8/15
Liver resection	1/0	2/3
SIRT and TACE	2/1	5/6
Other	2/2	5/5
**Neoadjuvant therapy**	**Number of patients outside the up to seven criteria** **(at the time of pre-transplant evaluation)/(based on postoperative histopathological findings)**	**Number of patients inside the up to seven criteria** **(at the time of pre-transplant evaluation)/(based on postoperative histopathological findings)**
None	6/9	11/8
TACE	6/6	43/43
SIRT	14/7	10/17
Liver resection	1/0	2/3
SIRT and TACE	2/1	5/6
Other	2/2	5/5

Abbreviations: SIRT: selective internal radiation therapy; TACE: Transarterial chemoembolization; UCSF: University of California San Francisco.

**Table 4:** Impact of Transplantation Criteria on Overall Survival.

	Number of Patients	OS (%)	*p*
**MILAN (Figure 4)**			0.014
No SIRT and no TACE	34	29.5	
TACE inside milan	37	82.7	
SIRT inside milan	6	83.3	
TACE outside milan	12	60	
SIRT outside milan	18	76.4	
**UCSF (Figure 5)**			0.002
No SIRT and no TACE	34	29.5	
TACE inside UCSF	41	85.6	
SIRT inside UCSF	8	75	
TACE outside UCSF	8	31.3	
SIRT outside UCSF	16	80.2	
**Up to seven (Figure 6)**			0.001
No SIRT and no TACE	34	29.5	
TACE inside up to seven	43	85.3	
SIRT inside up to seven	10	80	
TACE outside up to seven	6	33.3	
SIRT outside up to seven	14	77.9	

**Table 5:** Multivariate Analysis.

	Multivariate Analysis		
**Variables**	HR	95%CI	*p*-value
**Transplantation (LDLT, DDLT)**	1.706	0.554–5.253	0.352
**Steatosis**	2.101	0.255–17.283	0.490
**Blood type**	0.954	0.605–1.504	0.839
**AFP negative (at initial diagnosis)**	0.472	0.161–1.387	0.172
**AFP negative after neoadjuvant therapy**	2.190	1.047–4.579	0.037
**AFP negative postoperatively**	5.401	1.596–18.276	0.007
**Liver Cirrhosis**	0.416	0.045–3.839	0.439
**Neoadjuvant therapy**	1.450	1.101–1.908	0.008
**Child score**	0.936	0.539–1.624	0.813
**Response to neoadjuvant therapy**	3.592	1.461–8.834	0.005
**T Stage**	0.959	0.557–1.650	0.880
**Age at the time of liver transplantation**	1.014	0.952–1.081	0.658
**Gender**	0.864	0.185–4.039	0.853

## Data Availability

The data that support the findings of this study are not openly available due to reasons of sensitivity and are available from the corresponding author upon reasonable request.
